# Dr. White’s Paper

**Published:** 1887-10

**Authors:** 


DR. WHITE'S PAPER will be read with interest; it is one of the best accounts of the great event that has yet appeared, and does the Doctor credit. It is to be followed by others, giving account of the section work. The Doctor made the acquaintance of many of the foreign delegates, and speaks in high terms of their social qualities.

